# Domestic abuse in the Covid-19 pandemic: measures designed to overcome common limitations of trend measurement

**DOI:** 10.1186/s40163-023-00190-7

**Published:** 2023-06-13

**Authors:** Sarah Hodgkinson, Anthony Dixon, Eric Halford, Graham Farrell

**Affiliations:** 1 School of Criminology, University of Leicester, Leicester, UK; 2 School of Law, University of Leeds, Leeds, UK; 3 Rabdan Academy, Abu Dhabi, United Arab Emirates

**Keywords:** Domestic abuse, Intimate partner violence, Coronavirus, Change-point analysis, Anomaly detection, NLP

## Abstract

Research on pandemic domestic abuse trends has produced inconsistent findings reflecting differences in definitions, data and method. This study analyses 43,488 domestic abuse crimes recorded by a UK police force. Metrics and analytic approaches are tailored to address key methodological issues in three key ways. First, it was hypothesised that reporting rates changed during lockdown, so natural language processing was used to interrogate untapped free-text information in police records to develop a novel indicator of change in reporting. Second, it was hypothesised that abuse would change differentially for those cohabiting (due to physical proximity) compared to non-cohabitees, which was assessed via a proxy measure. Third, the analytic approaches used were change-point analysis and anomaly detection: these are more independent than regression analysis for present purposes in gauging the timing and duration of significant change. However, the main findings were largely contrary to expectation: (1) domestic abuse did not increase during the first national lockdown in early 2020 but increased across a prolonged post-lockdown period, (2) the post-lockdown increase did not reflect change in reporting by victims, and; (3) the proportion of abuse between cohabiting partners, at around 40 percent of the total, did not increase significantly during or after the lockdown. The implications of these unanticipated findings are discussed.

**Supplementary Information:**

The online version contains supplementary material available at 10.1186/s40163-023-00190-7.

## Background

The movement of people changed dramatically as restrictions were introduced to stem the spread of Covid-19 in early 2020. These health policies inadvertently transformed the crime opportunity structure via different mechanisms for different crime types with, generally speaking, many physical crime types declining dramatically (Ashby, 2020; Halford et al, 2020; Langton et al., 2021) while online crimes increased (Buil-Gil et al., 2021; Johnson & Nikolovska, 2022).

Domestic abuse is a hidden crime that often goes unreported to police or other agencies (Stanko 1988). A variety of sources, including others described further below, identified domestic abuse as a significant issue early in the pandemic (Kaukinen, 2020; Van Gelder et al., 2020), some suggesting major increases that comprised a shadow pandemic (UN Women, 2020) or an ‘epidemic beneath a pandemic’ (BBC, 2021). It is fair to suggest there was consensus that domestic abuse would increase with movement restrictions, particularly during stay-at-home lockdowns, consistent with lifestyle and routine activity theory and, more specifically, Halford et al.,’s (2020) mobility theory of crime change during the pandemic. This proposed that pandemic crime rates changed primarily due to changes in lifestyles and movement, specifically the rates of interaction of suitable targets and offenders in the absence of capable guardians due to the health policy restrictions. In relation to domestic abuse, the theory would predict that crime opportunities increased as partners were legally obliged to spend far more time together during stay-at-home lockdown, increasing the frequency of interaction in which abuse could occur, while reducing potential guardianship from non-cohabiting family and friends and reducing access to other domestic abuse services. In addition, the proportion of abuse by cohabiting partners would be expected to increase, reflecting absolute increases but also an increase relative to abuse from non-cohabitees with reduced access. It would also be expected that movement restrictions and social distancing would reduce the reporting and recording of domestic abuse through at least two mechanisms: the capacity of victims to report a crime would decrease if offenders remained in the household, and police would be less likely to record domestic abuse if they were unable to enter households to investigate. Victims were *trapped* with their abusers during lockdown (Johnson & Hohl, 2021), during lockdown, and perhaps less able to report abuse to police but more likely to report to other domestic abuse services (Women’s Aid, 2020; ONS, 2020a, 2020b).

The issues described above informed the hypotheses for this study; First, the rate of reporting of domestic abuse would decline during lockdown and subsequently resume; Second, change to domestic abuse rates would reflect differential change to that between cohabiting partners from that to non-cohabiting partners, particularly during lockdown, and; Third, domestic abuse rates would increase overall but particularly during lockdown.

### Previous studies

Research into domestic abuse during the pandemic has produced considerable variation in its findings. With respect to the UK, the Office for National Statistics concluded that “it is not possible to determine what impact the coronavirus pandemic may have had on the increases in 2020” (ONS 2020a; 5). This conclusion was based on the fact that, while there was a 7 percent increase in police-recorded domestic abuse crimes between March and June 2020 (during the first national lockdown), this was a continuation of the pre-pandemic trend which reflected increased reporting and recording (ONS 2020a, 2020b, 2021). Other aspects of the ONS reports assessed changes in volumes of calls to helplines and domestic abuse services in 2020, but these could not be distinguished from seasonal change and other factors. Findings from the Crime Survey for England and Wales were unavailable because face-to-face interviews were suspended from March 2020 to October 2021 (ONS 2022; 3).

It is perhaps surprising that the ONS reports concluded that it was not possible to determine the impact of the pandemic from police-recorded crime. It appears to contradict the finding that the pandemic trend continues the pre-pandemic trend, which would usually be interpreted as showing no evidence of a pandemic effect. If the ONS reports played to the gallery by fudging the issue, this could suggest confirmation bias (that is, an emphasis on the expected finding or on avoiding an unexpected finding). Perhaps the key issue is the role of possible changes to the reporting of domestic abuse: the general upheaval caused by the pandemic would make it easy to dismiss studies using police-recorded domestic abuse crimes, hospital records, or agency records, as insensitive to change in reporting to the police. It is for that reason that the present study develops an indicator of change in reporting.

In other UK-based work, Shepherd et al. (2020) compared pre- and pandemic hospital admissions records, finding that non-domestic violent injuries declined significantly but those within the home did not. Demands for domestic abuse services were found to increase during the early pandemic (ONS, 2020a, 2020b), with variation by geographic region (Speed et al., 2020). Online self-report surveys found demand for support services (for domestic abuse) had increased, the severity of abuse had worsened, and abusers made use of Covid-19 restrictions to control and frighten their victims (Women’s Aid, 2020, 2021).

A study of one UK police force identified a significant early-pandemic decrease in police-recorded domestic abuse relative to what would be expected based on previous years’ trends and controlling for seasonal variation (Halford et al., 2020). In contrast, an assessment of Metropolitan Police recorded crime data, comparing observed and expected levels (also using 5 years of data) to June 2020, found that abuse increased for victims living in the same household as their abuser (partners and family members) but decreased for those who were not, such as ex-partners (Ivandic et al., 2020). An analysis of domestic abuse recorded by seven UK police forces found increases were, similar to the ONS findings, largely attributable to pre-existing long-term trends (Johnson & Hohl, 2021).

Significant variation in findings is evident in the international literature. Studies focusing on the US have tended to find increased domestic abuse but with variation between states and cities (Nix & Richards, 2021), spanning evidence from hospital records of violent injuries at home (Gosangi et al., 2021), police calls for service (Mohler et al, 2020), police crime data (Evans et al., 2021; Hsu & Henke, 2021), and calls to domestic abuse charities and helplines (Sorensen et al. 2021). Increased domestic abuse was found to be of short duration, soon returning to pre-pandemic levels (Leslie & Wilson, 2020; McCrary and Sanga, 2020). Similar increases were found for India (Ravindran & Shah, 2020) and Argentina (Perez-Vincent et al., 2020). However it was suggested that measurement difficulties mean some studies could over-state findings (Reingle Gonzalez et al., 2020), also potentially reflecting confirmation bias. An early-pandemic UN report found mixed results but did not have the longer-term data of some studies that allowed for a more rigorous assessment (UNODC, 2020). Where significant increases in domestic abuse were identified using police data, the focus tended to be a short timespan at the pandemic’s onset, without necessarily accounting for pre-pandemic trends or seasonal variation (Payne et al., 2020).

Time series analysis of crime data which controlled for long-term and seasonal trends found that domestic abuse rates remained largely stable (Ashby, 2020; Campedelli et al., 2020; Reingle-Gonzalez et al., 2020). Payne et al. (2020) analysed breaches of domestic violence orders in Australia and found that, compared to expected levels (those expected in the absence of the pandemic) there was no significant change. Similar results were found in Sweden (Gerrell et al., 2020) and Mexico (de la Miyar et al., 2020). In the US, Piquero et al. (2020) found short-term ‘spikes’ immediately after lockdown in Dallas, but no significant change overall, other than increases consistent with the year-on-year pre-pandemic trend. Nix and Richards (2021) found a similar pattern in some US cities, but very different patterns in others. Bullinger et al. (2021) highlighted the differences between domestic-abuse related calls for service (which increased) and crimes and arrests (which fell).

A meta-analysis of 18 international studies identified an 8 percent increase in domestic violence, concluding there was evidence of an ‘overwhelming increase’ in domestic violence (Piquero et al., 2021). However, while most studies showed increases, there were some notable decreases. Elsewhere it was argued that analysing short time periods, as undertaken in many of the studies to that date, could either mask or exaggerate effects (Payne et al., 2020). Studies were also difficult to compare as they often differed in their definitions, methods, and data source.

It is difficult to distinguish the extent to which differences in findings between studies reflect variation in domestic abuse or variations in definitions, data sources, measurement, and other aspects of method. Cross-national comparison is made difficult by an absence of international consensus on what constitutes domestic abuse (Piquero et al., 2021). For instance, North American studies tend to prefer the term ‘domestic violence’, with an emphasis on physical violence whereas, in the UK the statutory definition of ‘domestic abuse’ since 2012 has included threatening, controlling, and coercive behaviour involving those aged over-16. Concerns that non-physical abuse is minimised by police have been suggested to be more of an issue in the US than UK (Robinson et al., 2016), and while some studies focus on ‘intimate partner violence’, others include child abuse and sibling-on-sibling incidents.

Differences between studies may also reflect differences between data source (Anderberg et al., 2020; Bougault et al., 2021). Police recorded crime data is often the most readily available data that spans the number of years preferred for predictive modelling of the rates that would be expected absent a pandemic. Yet recorded crime data is not necessarily comparable due to the definitions discussed above but also differences in recording practices between jurisdictions. The variety of other data sources can all differ significantly and include: surveys and self-report data; practitioner, administrative and public health records; breaches of existing domestic violence orders; calls for service; helpline data; or other violent crime data used as a proxy for domestic abuse. Whilst all of these sources can provide valuable indicators their inherent differences make comparison difficult.

### Timeline of UK pandemic regulations

To set the scene, this paragraph gives an overview of UK pandemic regulatory changes. The World Health Organisation (WHO) declared Covid-19 a pandemic on 11 March 2020, and Europe the epicentre on 13 March. Following a week where it was recommended that non-essential travel was avoided, the first UK national lockdown began 23 March with a legal requirement to stay-at-home aside from essential work and shopping, with no mixing outside of households. Measures were gradually relaxed in England and Wales from 10 May 2020, and on 1 June 2020 the Government introduced the ‘rule of six’ whereby groups of up to six people from more than one household could meet outdoors. Over the summer months, restrictions were further relaxed aside from local lockdowns for areas with high infection rates. In October 2020, restrictions were generally tightened but using a tiered-system of regional restrictions reflecting variations in infection rates. On 5 November 2020 the second national lockdown began, easing somewhat in early December, but high infection rates led to further restrictions for the late December holiday period. The third national lockdown ran from early January 2021 until 8 March 2021 when schools began to reopen (Barber et al., 2021).

## Method

### Data

Domestic abuse is defined under UK law as abusive behaviour by a person aged over 16 towards to a person who is personally connected to them, with abuse encompassing physical or sexual abuse; violent or threatening behaviour; controlling or coercive behaviour; economic abuse; psychological, emotional, or other abuse^1^. The data used here is domestic abuse crimes recorded by police following UK crime recording standards (Home Office 2022).

The study area was a provincial English police force serving nearly 1.5 million residents across roughly 1200 square miles (3108 square kilometres) with several small cities but predominately towns interspersed with large rural areas. Domestic abuse crimes were identified by a ‘Domestic abuse code’ or ‘flag’ in the police record. There were 43,488 domestic abuse crimes in total. The crime records included metadata on time, date and location of the crime and its reporting, offence code and victim-offender relationship. In addition, the police force provided unstructured Modus Operandi (MO) data, which was typically one or two sentences of free text comprising the reporting police officer’s brief description of the crime. The median number of words in an MO was 22, the 25th and 75th percentiles of words per MO were 15 and 33 respectively, and examples are shown in Table 1.

**Table 1:** Example of modus operandi free text

Example 1: Suspect has hit victim to the head with a staircase spindle
Example 2: KNOWN OFFENDER HAS APPROACHED THE VICTIM FROM BEHIND AND USED A SHARP OBJECT CAUSING A NUMBER OF MINOR LACERATIONS TO HIS FACE CAUSING MINOR INJURIES

The police force changed aspects of its crime recording system in late 2018. As this was likely to have disrupted the consistency of the data, the present study uses data for the two calendar years 2019 and 2020. This had implications for the analytic approach as described below.

The structured police records were aggregated to weekly crime counts. Weekly counts were preferred because daily data can be ‘noisier’ and more likely confounded by other variables. From here, the percentage of flagged domestic abuse crimes involving former and current partners was calculated.

### Natural language processing to develop the indicator of reporting

The reporting of multiple incidents by domestic abuse victims/survivors was estimated from the MO free texts using natural language processing (NLP) which quantified the extracted information. The NLP method, described further below, labelled the domestic abuse MO texts according to whether a single or multiple incidents were recorded. The national crime recording standards state that “an incident comprising a sequence of crimes between the same offender and the same victim should be counted as one crime if reported to the police all at once” (Home Office, 2022). However, the number of crimes reported was not separately recorded which is why it was sought from the MO free texts. The police records typically included one date for each crime, but when multiple crimes were reported, dates were sometimes recorded that spanned several days, weeks or months. This is consistent with the frequently repeated, ongoing or chronic nature of domestic abuse crimes which can span stalking and harassment, coercive control and false imprisonment. To overcome this limitation, the free text MOs were coded (labelled) to determine if each recorded crime included more than one incident of domestic abuse. If reporting to police declined during the first national lockdown and resumed afterwards, then this would be evidenced as an increase in the post-lockdown reporting of multiple incidents.

The NLP model used to classify the free-text was built using BERT pre-trained language models which has been used to classify short texts relating to other topics (Devlin et al., 2018). A finetuning process (Devlin et al., 2018) was used to train the model. Of the 43,488 MO texts, 1200 (2.7%) were read and manually labelled by two researchers to indicate whether they were single or multiple incidents. This sample was identified through an active learning process (Settles, 2012).

Data coding, known as labelling in relation to NLP, comprised reading the MO free text and assessing whether it described a single or multiple incidents of domestic abuse. For most records this was a straightforward exercise: single incidents were identifiable by phrases such as *‘hit the victim once’*, and multiple incidents were identified by phrases such as ‘*over the period of several days the victim was subject to physical abuse’*. Each crime was coded by two researchers, in the event of disagreement one of the authors had the casting vote. The most common source of ambiguity was when the time and date of the crime spanned a significant period of time. For example, if physical abuse occurred on two separate days this was evidence of multiple events. However, if abuse occurred on the same day but separated by several hours, it could be unclear whether it should be coded as one or multiple incidents, and these sometimes required adjudication. In general, a *‘finished incident’* rule was used to help determine whether multiple incidents occurred. This used information such as text describing a period of several hours between incidents, when incidents were separated by non-abuse activity, or information such as the offender leaving to go to work. Coding of free text data is a common research procedure and not without limitations. At minimum it provides a more accurate picture than the original recorded crime counts.

Once labelling was complete, 1000 of the 1200 manually labelled MOs were used to fine tune the BERT algorithm for the classification model. The BERT model was fine-tuned in Python 3.8 using the transformers package on a Pytorch framework (Wolf et al., 2020). The fine-tuned models were tested on the remaining human-labelled data (previously unseen by the model), and its performance judged using the F1 score. F1 is an appropriate measure for classification models with an unbalanced data set (i.e. one classification is significantly more common than the others), as here. An F1 score of 1 is perfect and a score of 0 is equivalent to random assignment. The present model achieved an F1 score of 0.84, there is no recognised benchmark to be achieved with F1 scores. However, as a comparison, human performance across a well-known set of NLP tasks (GLUE) is assessed to be 0.87 (Nangia & Bowman, 2019).

### Analytic approach

Change-point analysis and anomaly detection were selected as analytic approaches with inherent advantages for this research context. In particular, they overcome potential confirmation bias. Confirmation bias means researchers tend to find data patterns that they expect to find, particularly when an explanation for the change is assumed to be correct (Kahneman, 2011). It was suggested earlier that some studies of domestic abuse in the pandemic had been suggested to contain elements of confirmation bias. This potential bias is, we suggest, overcome here because change-point analysis and anomaly detection approaches both produce a test statistic that gauges whether there was statistically significant change. Hence, unlike interrupted time series analysis, there is no requirement for *a priori* specification of the timing or duration of expected change. In effect, the algorithms automate those decisions and thereby remove some of the subjectivity from the analysis. Both change-point analysis and anomaly detection have, to date, been infrequently utilised approaches for crime data (see Monyeki et al., 2020 for an exception) despite their advantages. Further, the two approaches are complementary and increased confidence in findings can be gained through their cross validation. In addition, it was not feasible to undertake ARIMA modelling for the present study as it requires more extensive historical data.

Change-point analysis identifies significant change in the mean or variance of time series data (Aminikhanghahi & Cook, 2017). Anomaly detection assumes an underlying time series with single or multiple joined time periods with different underlying characteristics – typically either a change in the time series mean or variance (Fisch et al., 2020). These differences comprise the anomaly. Note that an important feature is how the algorithm expects a time series to return to the’normal structure’ after the anomaly.

#### Change-point analysis

The MOSUM package (Meier et al., 2021a, 2021b) proposed in Eichinger and Kirch (2018) and Cho and Kirch (2021) was adopted because its theoretical consistency is established under general conditions that accommodate the characteristics observed in the present dataset. Specifically, the threshold for the test statistic was generated through simulated data based on the mean and standard deviation of the 2019 data (see code in Additional file 1). The MOSUM package gauges change by computing a test statistic drawn from different subsections of the data. The size of the subsection in each step is determined by the bandwidth provided to the main algorithm. The test statistic increases when a change is detected in the data, and a change is marked as significant when the test statistic crosses a pre-determined threshold. In the present analysis the threshold value was computed by randomly simulating data with 2019 characteristics (i.e. mean and variation—see code for exact procedure.) and selecting the upper 95% quantile of the maximum test statistic from each simulation, thus highlighting changes at the 5% significance level Additional file 2.

The simulations used here drew on data from 2019 and therefore preceded the pandemic. The lower bandwidth was set at a value of 2 so that it included changes relatively close to one another. Following Meier et al., (2021a, 2021b), and in order that it was no more than four times the lower value, the upper bandwidth was set at 8. Also following Meier et al., (2021a, 2021b), the multi-bandwidth MOSUM pruning algorithm was used because the lower bandwidth was considered small (Meier et al., 2021a, 2021b). Due to the importance of bandwidth selection, sensitivity analysis around it was undertaken (see Table 2). Note that the values in the bandwidth represent a range rather than two single values.

**Table 2:** Changepoints analysis results (including sensitivity analysis to vary bandwidth)

Weekly metric	Bandwidth	Changepoint dates	Interpretation
All domestic abuse crime	2:6	2019-12-15	Increase during late-Dec holiday period
		2019-12-29	
	2:8	2019-12-15	(duplicate)
		2019-12-29	
		2020-06-14	Post-lockdown increase
		2020-08-30	
	4:20	2019-12-15	(duplicate)
		2019-12-29	
		2020-03-01	Early-pandemic decrease
		2020-04-12	
		2020-06-14	(duplicate)
		2020-08-30	
	10:40	2019-12-15	Duplicate (slightly extended duration)
		2020-03-01	
		2020-04-12	No obvious interpretation
		2020-06-14	Duplicate (slightly extended duration)
		2020-08-09	
Former partner	2:6	None detected	No significant differences between current and former partners
	2:8	None detected	
	4:20	2019-08-25	Some pre-pandemic difference detected at higher bandwidths
		2019-11-17	
		2019-12-22	
	10:40	2019-08-25	
Reporting multiple events	2:6	None detected	No significant changes in reporting detected
	2:8	None detected	
	4:20	None detected	
	10:40	None detected	

Changepoints identified during analysis and sensitivity analysis

#### Anomaly detection

The collective and point anomalies (CAPA) algorithm (Fisch et al., 2018) from the R package ‘Anomaly’ (Fisch et al., 2020) was adopted because it has the capacity to detect collective anomalies (periods of change in the crime data) rather than solely single instances. The CAPA algorithm requires selection of the minimum segment length to be detected and ‘Beta’. ‘Beta’ is the penalty value to which the test statistic is compared. A value of 2 was used here as the minimum segment length so that short periods (i.e. two consecutive weeks) of change were identified. For the Beta value, the default recommended value which proved effective for anomaly detection with the CAPA algorithm was retained (Fisch et al., 2018).

## Results

**Fig. 1:**
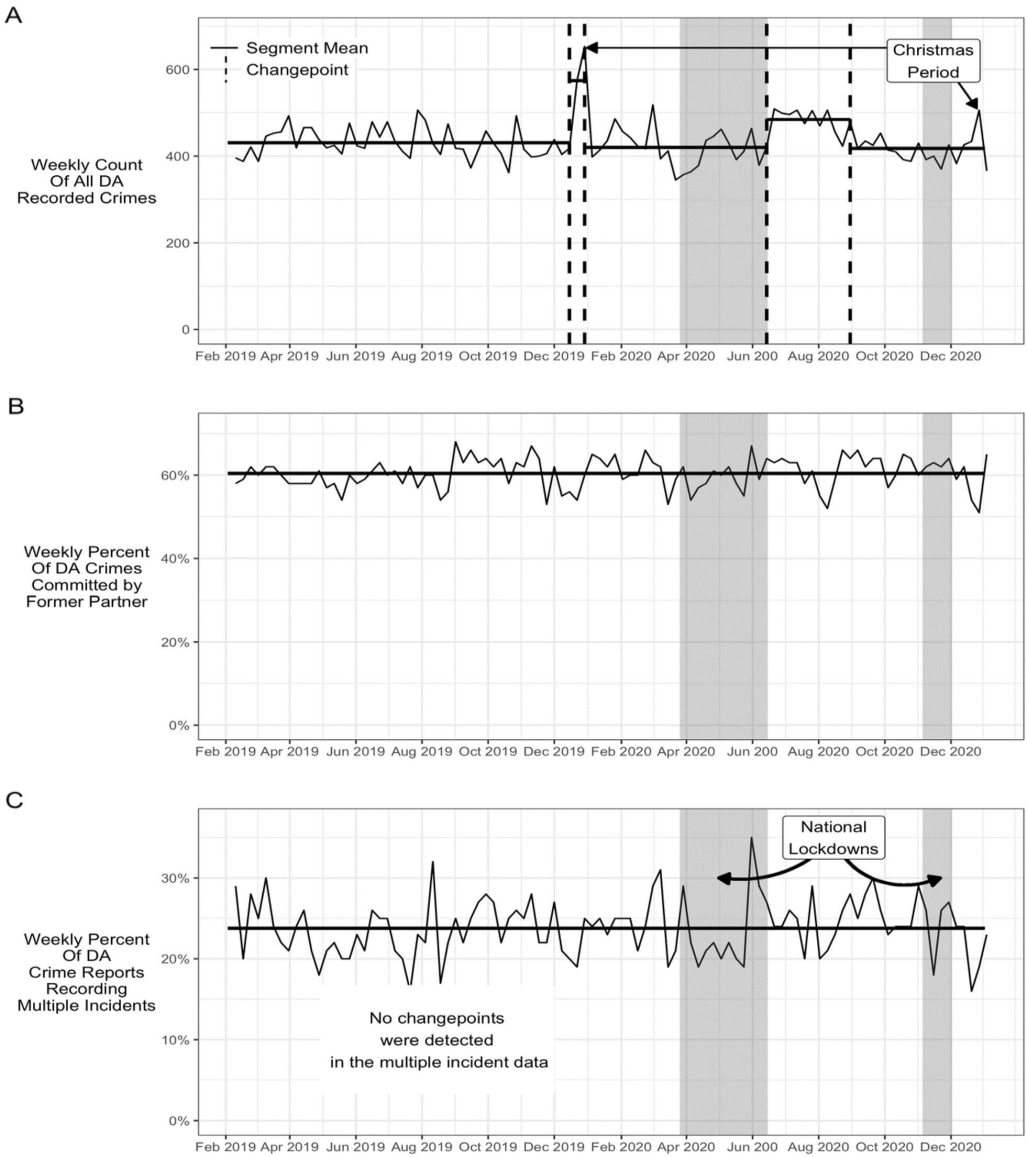
Weekly recorded domestic abuse crimes: Panel **A** All recorded domestic abuse crimes; Panel **B** Percentage committed by former partner; Panel **C** Percentage identified as multiple incident crimes

The main results are shown as Fig. 1 which incorporates three panels described in turn below. Figure 1 incorporates the identified change-point dates listed in Table 2, and Table 2 incorporates the results of the sensitivity analysis described below. In Panel A of Fig. 1, the dashed vertical lines indicate the change-points identified by the analysis. Note that no change-points were identified for the analyses in Panels B and C and so no vertical dashed lines are shown (discussed further below). The mean value of each segment is shown as a solid horizontal line in each panel, and each panel has the same shaded areas that span the dates of the two national lockdowns in 2020. Changes detected by the CAPA package were almost identical and so are not shown.

Figure 1 panel A shows the number of weekly domestic abuse crimes recorded in the study area. Change-points were identified for the late-December holiday periods of 2019 (an increase) and at the end of the first lockdown in June 2020 (an increase). Given that bandwidth is an important input to the MOSUM algorithm, a sensitivity analysis was undertaken which involved varying the bandwidth. The results are incorporated in Table 2. The sensitivity analysis did not alter the existing changepoints. However, it identified an *additional* changepoint comprising a four-week reduction during the first national lockdown. Evidence for this changepoint is weaker as it was identified only by extending the parameters, and it is not shown on Panel A for this reason.

In short, the results provide some evidence of a four-week increase during first lockdown, and strong evidence of a prolonged post-lockdown decline spanning July to the end of August 2020.

Panel B of Fig. 1 shows the percentage of domestic abuse crimes recorded as committed by former partners. The proportion of recorded domestic abuse crimes committed by former partners remained fairly constant at around 60 percent of the total, with variation around that mean essentially conforming to a random walk. That is, no statistically significant anomalies were detected, and no change-points were detected (except with the more relaxed parameters of the sensitivity analysis and then for 2019 only, that is, before the pandemic).

Figure. 1 Panel C shows the percentage of recorded DA crimes that were identified as including multiple incidents using NLP. The rate was typically around 24 percent of the total. No statistically significant changepoints were identified in the multiple-incident data by either the anomaly or the MOSUM package. However, Panel C does show a spike to around 40 percent in the final weeks of the first national lockdown. The spike would represent a two-thirds increase (that is, 40 compared to 24). However the increase was of short duration and, in the absence of any statistically significant difference, therefore cannot be conclusively identified as a pandemic-related effect^2^

## Discussion

It was no surprise to find a short-term increase in recorded domestic abuse crimes in the late-December 2019 holiday period. The identification of a statistically significant change at this time, before the pandemic, is methodologically reassuring because it provides a demonstration test of the analytic approach. It squares with the interpretation of increases in domestic abuse occurring when opportunities increase with the rate of interaction.

It was hypothesised that domestic abuse rates overall would increase during the first national lockdown, driven by increases between cohabitees. However, total recorded domestic abuse rates were found to decrease early in the pandemic, although this only became statistically significant when the model parameters were expanded during sensitivity analysis. A post-lockdown increase was also identified that lasted to the end of August 2020. These findings are contrary to expectation and tend towards the rejection of the hypothesis that domestic abuse would increase. While the ‘total’ recorded domestic abuse rate does not distinguish cohabitees and non-cohabitees, and recorded crime data can be subject to changes in reporting and recording, the other components of our analysis overcame those issues. 

The post-lockdown increase in domestic abuse identified here might be interpreted as similar to what occurred for many crime types including violence. In the post-lockdown period, the rates of many crime types increased relative to their reduced lockdown rates. The post-lockdown increases in violent crime and public disorder reflected increased interactions of persons in the public sphere once stay-at-home orders were removed. It is possible that increased public interactions had a similar effect upon domestic abuse, even though this seems contrary to the crime being ‘domestic’ in nature. It is easy to forget that domestic abuse is defined by relationships rather than by location.

The data on ‘current and former partners’ offered a proxy measure of cohabiting and non-cohabiting partners. The study found relatively stable proportions, and no significant change-points, in the rate of recorded domestic abuse by current and former partners across 2020. This tends towards rejection of the hypothesis that abuse increased more between cohabiting partners.

Changes to reporting could, in theory, explain the other findings. However, the indicator of change in reporting did not identify change: a slight increase in the reporting of multiple incidents was not statistically significant, and no statistically significant change-points in reporting were identified. This also tend towards rejection of the hypotheses that reporting of domestic abuse had changed, and supports the rejection of the other hypotheses.

These findings are potentially important. Unexpectedly, we found no evidence of increased domestic abuse overall during lockdown, no evidence of an increase in domestic abuse between current partners (and no evidence of a decrease between former partners), and no significant evidence of under-reporting during lockdown. Why? The two contending explanations are that the findings accurately gauge trends in domestic abuse, and the second is that they are artefacts of data and method. If the findings reflect reality then it is quite a different reality to what was expected, so let us turn first review possible limitations of the study as this will clarify its overall utility.

A known limitation of police records is that much domestic abuse goes unreported and unrecorded. However, trend analysis such as that undertaken here is reliable if it is reasonable to assume there is no change in the likelihood of reporting and recording. This is why an indicator of reporting change was developed for the present study. If our indicator is accurate then it suggests the trends in domestic abuse are reliable. The NLP indicator of single and multiple incident reporting was constrained by the quality of the information recorded in the MO free text fields. Quality control for the NLP labelling work was promoted here by the process of two researchers labelling each record independently, and the F1 score of 0.84 indicated that the accuracy of the NLP classifier was high. However, we acknowledge that further research could utilise more formal measures of inter-rater reliability, that the F1 score denotes some imperfect, and that replication and extension of the research would be informative.

Advantages of the analytic approach were outlined above, However, alternative analyses could have been undertaken. In particular, a regression model could compare recorded domestic abuse between 2019 and 2020 by controlling for month and year fixed effects, using binary indicator variables for lockdowns and periods of relaxed restrictions. Such analysis would likely be noisier, and seasonality across the week would be a potential confound in relation to the present dataset, while a key disadvantage of regression analysis would be the requirement to specify when changes took place, which was not a limitation of the approaches adopted here.

This further review of possible limitations leads us to conclude that the study is likely to give an accurate representation of what occured. If that is the case, what are the implications? It would mean that domestic abuse did not increase, that even domestic abuse between cohabiting partners did not increase, and that reporting of domestic abuse was not unduly affected. These findings runs contrary to theoretically-informed expectation and the suggestion of many other commentators. However, they would be consistent with our reinterpretation of the findings from the Office for National Statistics.

## Conclusion

Police recorded crime data have strengths and limitations for research. Key strengths derive from the fact they are routinely collected for operational purposes and so are more readily and cheaply available on a timelier basis than most other data. They can also provide information rarely available elsewhere including date, time, and geolocation of crime events, which is difficult to collect via victim surveys due to memory recall and other methodological issues. These strengths are balanced by the limitation of much crime going unreported and unrecorded. This is particularly the case for domestic abuse, for which only a relatively small proportion reaches crime records. Nevertheless, recorded crime data provide indicative trends if under-recording remains consistent. The novel indicator developed here using natural language processing suggested that reporting to police did not change.

The study's novel indicators and the analytic approach add to the repertoire of techniques that address key limitations of police recorded crime data, and the findings add to knowledge about the impact of the pandemic upon domestic abuse crime. Three key aspects of domestic abuse crime during the pandemic were explored: the extent of domestic abuse, the type of victim-offender relationship, and issues of delayed reporting during lockdown.

Contrary to expectation, we found a notable decrease in recorded domestic abuse crime in the early stages of the pandemic and the early part of the first national lockdown. However, after the first lockdown ended, there was an increase of close to 20 percent in recorded domestic abuse crimes which lasted until the end of August 2020. The net increase was greater than the earlier decrease, but these changes did not reflect change  in the reporting of domestic abuse by victims who were ‘trapped’ during the pandemic. In addition, and also contrary to expectation, there was no significant change in the proportion of domestic abuse by former or current partners.

## Supplementary Information

**Additional file 1.** Changepoint detection code.

**Additional file 2.** Plot of delay and duration data.

## Data Availability

The police data is restricted. Authors will provide details of data owner upon request. Code will be deposited in a code repository.

## Footnotes

1 https://www.legislation.gov.uk/ukpga/2021/17/section/1/enacted accessed 13 March 2022.

2 We used data on the recorded duration of the crime, and the time from when the crime occurred and when it was reported (i.e. any delay in reporting), and found no evidence that reporting was delayed. However, the duration and delay data was fragmented and generally poor: we suspect the time of the crime and the time of reporting was routinely recorded as the same, or that the data field was poorly completed. In short, there was no evidence that the spike in multiple incidents reflected delayed reporting.
