# Supplementary figures and images for: Domestic abuse in the Covid-19 pandemic: measures designed to overcome common limitations of trend measurement
Source: Crime Sci. 2023 Jun 13;12(1):12. doi: 10.1186/s40163-023-00190-7 (PMC10262932; doi:10.1186/s40163-023-00190-7)

**Additional Material**

**Plot of delay and duration data**


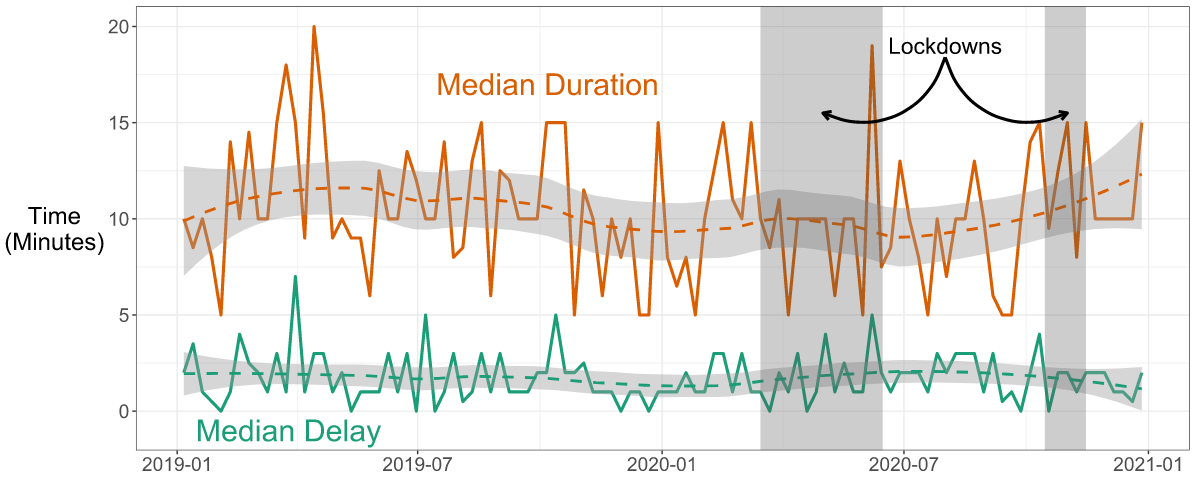

Supplement: Supplementary file 2 — Additional file 2. Plot of delay and duration data. [file 40163_2023_190_MOESM2_ESM.docx]
